# Artificial Intelligence in Education (AIEd): a high-level academic and industry note 2021

**DOI:** 10.1007/s43681-021-00074-z

**Published:** 2021-07-07

**Authors:** Muhammad Ali Chaudhry, Emre Kazim

**Affiliations:** 1 Artificial Intelligence at University College, London, UK; 2 Department of Computer Science, University College, London, UK

**Keywords:** Artificial Intelligence, Education, Machine learning, Learning science, Ethical AI, Artificial Intelligence in Education (AIEd), Intelligent Tutoring Systems (ITS), Fairness

## Abstract

In the past few decades, technology has completely transformed the world around us. Indeed, experts believe that the next big digital transformation in how we live, communicate, work, trade and learn will be driven by Artificial Intelligence (AI) [83]. This paper presents a high-level industrial and academic overview of AI in Education (AIEd). It presents the focus of latest research in AIEd on reducing teachers’ workload, contextualized learning for students, revolutionizing assessments and developments in intelligent tutoring systems. It also discusses the ethical dimension of AIEd and the potential impact of the Covid-19 pandemic on the future of AIEd’s research and practice. The intended readership of this article is policy makers and institutional leaders who are looking for an introductory state of play in AIEd.

## Introduction

Artificial Intelligence (AI) is changing the world around us [42]. As a term it is difficult to define even for experts because of its interdisciplinary nature and evolving capabilities. In the context of this paper, we define AI as a computer system that can achieve a particular task through certain capabilities (like speech or vision) and intelligent behaviour that was once considered unique to humans [54]. In more lay terms we use the term AI to refer to intelligent systems that can automate tasks traditionally carried out by humans. Indeed, we read AI within the continuation of the digital age, with increased digital transformation changing the ways in which we live in the world. With such change the skills and knowhow of people must reflect the new reality and within this context, the World Economic Forum identified sixteen skills, referred to as twenty-first century skills necessary for the future workforce [79]. This includes skills such as technology literacy, communication, leadership, curiosity, adaptability, etc. These skills have always been important for a successful career, however, with the accelerated digital transformation of the past 2 years and the focus on continuous learning in most professional careers, these skills are becoming necessary for learners.

AI will play a very important role in how we teach and learn these new skills. In one dimension, ‘AIEd’ has the potential to dramatically automate and help track the learner’s progress in all these skills and identify where best a human teacher’s assistance is needed. For teachers, AIEd can potentially be used to help identify the most effective teaching methods based on students’ contexts and learning background. It can automate monotonous operational tasks, generate assessments and automate grading and feedback. AI does not only impact what students learn through recommendations, but also how they learn, what are the learning gaps, which pedagogies are more effective and how to retain learner’s attention. In these cases, teachers are the ‘human-in-the-loop’, where in such contexts, the role of AI is only to enable more informed decision making by teachers, by providing them predictions about students' performance or recommending relevant content to students after teachers' approval. Here, the final decision makers are teachers.

Segal et al. [58] developed a system named SAGLET that utilized ‘human-in-the-loop’ approach to visualize and model students’ activities to teachers in real-time enabling them to intervene more effectively as and when needed. Here the role of AI is to empower the teachers enabling them to enhance students’ learning outcomes. Similarly, Rodriguez et al. [52] have shown how teachers as ‘human-in-the-loop’ can customize multimodal learning analytics and make them more effective in blended learning environments.

Critically, all these achievements are completely dependent on the quality of available learner data which has been a long-lasting challenge for ed-tech companies, at least until the pandemic. Use of technology in educational institutions around the globe is increasing [77], however, educational technology (ed-tech) companies building AI powered products have always complained about the lack of relevant data for training algorithms. The advent and spread of Covid in 2019 around the world pushed educational institutions online and left them at the mercy of ed-tech products to organize content, manage operations, and communicate with students. This shift has started generating huge amounts of data for ed-tech companies on which they can build AI systems. According to a joint report: ‘Shock to the System’, published by Educate Ventures and Cambridge University, optimism of ed-tech companies about their own future increased during the pandemic and their most pressing concern became recruitment of too many customers to serve effectively [15].

Additionally, most of the products and solutions provided by ed-tech start-ups lack the quality and resilience to cope with intensive use of several thousands of users. Product maturity is not ready for huge and intense demand as discussed in Sect. “Latest research” below. We also discuss some of these products in detail in Sect. “Industry’s focus” below. How do we mitigate the risks of these AI powered products and who monitors the risk? (we return to this theme in our discussion of ethics—Sect. “Ethical AIEd”).

This paper is a non-exhaustive overview of AI in Education that presents a brief survey of the latest developments of AI in Education. It begins by discussing different aspects of education and learning where AI is being utilized, then turns to where we see the industry’s current focus and then closes with a note on ethical concerns regarding AI in Education. This paper also briefly evaluates the potential impact of the pandemic on AI’s application in education. The intended readership of this article is the policy community and institutional executives seeking an instructive introduction to the state of play in AIEd. The paper can also be read as a rapid introduction to the state of play of the field.

## Latest research

Most work within AIEd can be divided into four main subdomains. In this section, we survey some of the latest work in each of these domains as case studies:

- Reducing teachers’ workload: the purpose of AI in Education is to reduce teachers’ workload without impacting learning outcomes
- Contextualized learning for students: as every learner has unique learning needs, the purpose of AI in Education is to provide customized and/or personalised learning experiences to students based on their contexts and learning backgrounds.
- Revolutionizing assessments: the purpose of AI in Education is to enhance our understanding of learners. This not only includes what they know, but also how they learn and which pedagogies work for them.
- Intelligent tutoring systems (ITS): the purpose of AI in Education is to provide intelligent learning environments that can interact with students, provide customized feedback and enhance their understanding of certain topics

### Reducing teachers’ workload

Recent research in AIEd is focusing more on teachers than other stakeholders of educational institutions, and this is for the right reasons. Teachers are at the epicenter of every learning environment, face to face or virtual. Participatory design methodologies ensure that teachers are an integral part of the design of new AIEd tools, along with parents and learners [45]. Reducing teachers’ workload has been a long-lasting challenge for educationists, hoping to achieve more affective teaching in classrooms by empowering the teachers and having them focus more on teaching than the surrounding activities.

With the focus on online education during the pandemic and emergence of new tools to facilitate online learning, there is a growing need for teachers to adapt to these changes. Importantly, teachers themselves are having to re-skill and up-skill to adapt to this age, i.e. the new skills that teachers need to develop to fully utilize the benefits of AIEd [39]. First, they need to become tech savvy to understand, evaluate and adapt new ed-tech tools as they become available. They may not necessarily use these tools, but it is important to have an understanding of what these tools offer and if they share teachers’ workload. For example, Zoom video calling has been widely used during the pandemic to deliver lessons remotely. Teachers need to know not only how to schedule lessons on Zoom, but also how to utilize functionalities like breakout rooms to conduct group work and Whiteboard for free style writing. Second, teachers will also need to develop analytical skills to interpret the data that are visualized by these ed-tech tools and to identify what kind of data and analytics tools they need to develop a better understanding of learners. This will enable teachers to get what they exactly need from ed-tech companies and ease their workload. Third, teachers will also need to develop new team working, group and management skills to accommodate new tools in their daily routines. They will be responsible for managing these new resources most efficiently.

Selwood and Pilkington [61] showed that the use of Information and Communication Technologies (ICT) leads to a reduction in teachers’ workload if they use it frequently, receive proper training on how to use ICT and have access to ICT in home and school. During the pandemic, teachers have been left with no options other than online teaching. Spoel et al. [76] have shown that the previous experience with ICT did not play a significant role in how they dealt with the online transition during pandemic. Suggesting that the new technologies are not a burden for teachers. It is early to draw any conclusions on the long-term effects of the pandemic on education, online learning and teachers’ workload. Use of ICT during the pandemic may not necessarily reduce teacher workload, but change its dynamics.

### Contextualized learning for students

Every learner has unique learning contexts based on their prior knowledge about the topic, social background, economic well-being and emotional state [41]. Teaching is most effective when tailored to these changing contexts. AIEd can help in identifying the learning gaps in each learner, offer content recommendations based on that and provide step by step solutions to complex problems. For example, iTalk2Learn is an opensource platform that was developed by researchers to support math learning among students between 5 and 11 years of age [22]. This tutor interacted with students through speech, identified when students were struggling with fractions and intervened accordingly. Similarly, Pearson has launched a calculus learning tool called AIDA that provides step by step guidance to students and helps them complete calculus tasks. Use of such tools by young students also raises interesting questions about the illusion of empathy that learners may develop towards such educational bots [73].

Open Learner Models [12, 18] have been widely used in AIEd to facilitate learners, teachers and parents in understanding what learners know, how they learn and how AI is being used to enhance learning. Another important construct in understanding learners is self-regulated learning [10, 68]. Zimmerman and Schunk [85] define self-regulated learning as learner’s thoughts, feelings and actions towards achieving a certain goal. Better understanding of learners through open learner models and self-regulated learning is the first step towards contextualized learning in AIEd. Currently, we do not have completely autonomous digital tutors like Amazon’s Alexa or Apple’s Siri for education but domain specific Intelligent Tutoring Systems (ITS) are also very helpful in identifying how much students know, where they need help and what type of pedagogies would work for them.

There are a number of ed-tech tools available to develop basic literacy skills in learners like double digit division or improving English grammar. In future, AIEd powered tools will move beyond basic literacy to develop twenty-first century skills like curiosity [49], initiative and creativity [51], collaboration and adaptability [36].

### Revolutionizing assessments

Assessment in educational context refers to ‘any appraisal (or judgement or evaluation) of a student’s work or performance’ [56]. Hill and Barber [27] have identified assessments as one of the three pillars of schooling along with curriculum and learning and teaching. The purpose of modern assessments is to evaluate what students know, understand and can do. Ideally, assessments should take account of the full range of student abilities and provide useful information about learning outcomes. However, every learner is unique and so are their learning paths. How can standardized assessment be used to evaluate every student, with distinct capabilities, passions and expertise is a question that can be posed to broader notions of educational assessment. According to Luckin [37] from University College London, ‘AI would provide a fairer, richer assessment system that would evaluate students across a longer period of time and from an evidence-based, value-added perspective’.

AIAssess is an example of an intelligent assessment tool that was developed by researchers at UCL Knowledge lab [38, 43]. It assessed students learning math and science based on three models: knowledge model, analytics model and student model. Knowledge component stored the knowledge about each topic, the analytics component analyzed students’ interactions and the student model tracked students’ progress on a particular topic. Similarly, Samarakou et al. [57] have developed an AI assessment tool that also does qualitative evaluation of students to reduce the workload of instructors who would otherwise spend hours evaluating every exercise. Such tools can be further empowered by machine learning techniques such as semantic analysis, voice recognition, natural language processing and reinforcement learning to improve the quality of assessments.

### Intelligent tutoring systems (ITS)

An intelligent tutoring system is a computer program that tries to mimic a human teacher to provide personalized learning to students [46, 55]. The concept of ITS in AIEd is decades old [9]. There have always been huge expectations from ITS capabilities to support learning. Over the years, we have observed that there has been a significant contrast between what ITS were envisioned to deliver and what they have actually been capable of doing [4].

A unique combination of domain models [78], pedagogical models [44] and learner models [20] were expected to provide contextualized learning experiences to students with customized content, like expert human teachers [26, 59, 65],. Later, more models were introduced to enhance students' learning experience like strategy model, knowledge-base model and communication model [7]. It was expected that an intelligent tutoring system would not just teach, but also ensure that students have learned. It would care for students [17]. Similar to human teachers, ITS would improve with time. They would learn from their experiences, ‘understand’ what works in which contexts and then help students accordingly [8, 60].

In recent years, ITS have mostly been subject and topic specific like ASSISTments [25], iTalk2Learn [23] and Aida Calculus. Despite being limited in terms of the domain that a particular intelligent tutoring system addresses, they have proven to be effective in providing relevant content to students, interacting with students [6] and improving students’ academic performance [18, 41]. It is not necessary that ITS would work in every context and facilitate every teacher [7, 13, 46, 48]. Utterberg et al. [78] showed why teachers have abandoned technology in some instances because it was counterproductive. They conducted a formative intervention with sixteen secondary school mathematics teachers and found systemic contradictions between teachers’ opinions and ITS recommendations, eventually leading to the abandonment of the tool. This highlights the importance of giving teachers the right to refuse AI powered ed-tech if they are not comfortable with it.

Considering a direct correlation between emotions and learning [40] recently, ITS have also started focusing on emotional state of students while learning to offer a more contextualized learning experience [24].

### Popular conferences

To reflect on the increasing interest and activity in the space of AIEd, some of the most popular conferences in AIEd are shown in Table 1 below. Due to the pandemic all these conferences will be available virtually in 2021 as well. The first international workshop on multimodal artificial intelligence in education is being organized at AIEd [74] conference to promote the importance of multimodal data in AIEd.

**Table 1:** Details of conferences relevant for Artificial Intelligence in Education

Conference name	Organizer	Locations
Artificial Intelligence in Education (AIEd)	International Artificial Intelligence in Education Society	Different locations every year, 2021 conference will be in Utrecht (The Netherlands) and virtual
Learning Analytics and Knowledge (LAK)	Society for Learning Analytics and Research	Different locations every year, 2021 conference will be virtual
Conference on Human Factors in Computing Systems	Association for Computing Machinery (ACM)	In Yokohama, Japan. 2021 conference will be virtual too

Table 1 shows some notable conferences that focus on Artificial Intelligence in Education. However, considering the interdisciplinary nature of AIEd, it is common to find relevant research being published in other AI and learning science conferences like Neurips, ICLR, ICML, ICLS and FAccT

## Industry’s focus

In this section, we introduce the industry focus in the area of AIEd by case-studying three levels of companies start-up level, established/large company and mega-players (Amazon, Cisco). These companies represent different levels of the ecosystem (in terms of size).

### Start-ups

There have been a number of ed-tech companies that are leading the AIEd revolution. New funds are also emerging to invest in ed-tech companies and to help ed-tech start-ups in scaling their products. There has been an increase in investor interest [21]. In 2020 the amount of investment raised by ed-tech companies more than doubled compared to 2019 (according to Techcrunch). This shows another dimension of pandemic’s effect on ed-tech. With an increase in data coming in during the pandemic, it is expected that industry’s focus on AI powered products will increase.

EDUCATE, a leading accelerator focused on ed-tech companies supported by UCL Institute of Education and European Regional Development Fund was formed to bring research and evidence at the centre of product development for ed-tech. This accelerator has supported more than 250 ed-tech companies and 400 entrepreneurs and helped them focus on evidence-informed product development for education.

Number of ed-tech companies are emerging in this space with interesting business models. Third Space Learning offers maths intervention programs for primary and secondary school students. The company aims to provide low-cost quality tuition to support pupils from disadvantaged backgrounds in UK state schools. They have already offered 8,00,000 h of teaching to around 70,000 students, 50% of who were eligible for free meals. Number of mobile apps like Kaizen Languages, Duolingo and Babbel have emerged that help individuals in learning other languages.

### Established players

Pearson is one of the leading educational companies in the world with operations in more than 70 countries and more than 22,000 employees worldwide. They have been making a transition to digital learning and currently generate 66% of their annual revenue from it. According to Pearson, they have built world’s first AI powered calculus tutor called Aida which is publicly available on the App Store. But, its effectiveness in improving students’ calculus skills without any human intervention is still to be seen.

India based ed-tech company known for creating engaging educational content for students raised investment at a ten billion dollar valuation last year [70]. Century tech is another ed-tech company that is empowering learning through AI. They claim to use neuroscience, learning science and AI to personalize learning and identifying the unique learning pathways for students in 25 countries. They make more than sixty thousand AI powered smart recommendations to learners every day.

Companies like Pearson and Century Tech are building great technology that is impacting learners across the globe. But the usefulness of their acclaimed AI in helping learners from diverse backgrounds, with unique learning needs and completely different contexts is to be proven. As discussed above, teachers play a very important role on how their AI is used by learners. For this, teacher training is vital to fully understand the strengths and weaknesses of these products. It is very important to have an awareness of where these AI products cannot help or can go wrong so teachers and learners know when to avoid relying on them.

In the past few years, the popularity of Massive Online Open Courses (MOOCS) has grown exponentially with the emergence of platforms like Coursera, Udemy, Udacity, LinkedIn Learning and edX [5, 16, 28]. AI can be utilized to develop a better understanding of learner behaviour on MOOCS, produce better content and enhance learning outcomes at scale. Considering these platforms are collecting huge amounts of data, it will be interesting to see the future applications of AI in offering personalized learning and life-long learning solutions to their users [81].

### Mega-players

Seeing the business potential of AIEd and the kind of impact it can have on the future of humanity, some of the biggest tech companies around the globe are moving into this space. The shift to online education during the pandemic boosted the demand for cloud services. Amazon’s AWS (Amazon Web Services) as a leader in cloud services provider facilitated institutions like Instituto Colombiano para la Evaluacion de la Educacion (ICFES) to scale their online examination service for 70,000 students. Similarly, LSE utilized AWS to scale their online assessments for 2000 students [1, 3].

Google’s CEO Sunder Pichai stated that the pandemic offered an incredible opportunity to re-imagine education. Google has launched more than 50 new software tools during the pandemic to facilitate remote learning. Google Classroom which is a part of Google Apps for Education (GAFE) is being widely used by schools around the globe to deliver education. Research shows that it improves class dynamics and helps with learner participation [2, 29, 62, 63, 69].

Before moving onto the ethical dimensions of AIEd, it is important to conclude this section by noting an area that is of critical importance to processing industry and services. Aside from these three levels of operation (start-up, medium, and mega companies), there is the question of development of the AIEd infrastructure. As Luckin [41] points out, “True progress will require the development of an AIEd infrastructure. This will not, however, be a single monolithic AIEd system. Instead, it will resemble the marketplace that has been developed for smartphone apps: hundreds and then thousands of individual AIEd components, developed in collaboration with educators, conformed to uniform international data standards, and shared with researchers and developers worldwide. These standards will enable system-level data collation and analysis that help us learn much more about learning itself and how to improve it”.

## Ethical AIEd

With a number of mishaps in the real world [31, 80], ethics in AI has become a real concern for AI researchers and practitioners alike. Within computer science, there is a growing overlap with the broader Digital Ethics [19] and the ethics and engineering focused on developing Trustworthy AI [11]. There is a focus on fairness, accountability, transparency and explainability [33, 82–84]. Ethics in AI needs to be embedded in the entire development pipeline, from the decision to start collecting data till the point when the machine learning model is deployed in production. From an engineering perspective, Koshiyama et al. [35] have identified four verticals of algorithmic auditing. These include performance and robustness, bias and discrimination, interpretability and explainability and algorithmic privacy.

In education, ethical AI is crucial to ensure the wellbeing of learners, teachers and other stakeholders involved. There is a lot of work going on in AIEd and AI powered ed-tech tools. With the influx of large amounts of data due to online learning during the pandemic, we will most likely see an increasing number of AI powered ed-tech products. But ethics in AIEd is not a priority for most ed-tech companies and schools. One of the reasons for this is the lack of awareness of relevant stakeholders regarding where AI can go wrong in the context of education. This means that the drawbacks of using AI like discrimination against certain groups due to data deficiencies, stigmatization due to reliance on certain machine learning modelling deficiencies and exploitation of personal data due to lack of awareness can go unnoticed without any accountability.

An AI wrongly predicting that a particular student will not perform very well in end of year exams or might drop out next year can play a very important role in determining that student’s reputation in front of teachers and parents. This reputation will determine how these teachers and parents treat that learner, resulting in a huge psychological impact on that learner, based on this wrong description by an AI tool. One high-profile case of harm was in the use of an algorithm to predict university entry results for students unable to take exams due to the pandemic. The system was shown to be biased against students from poorer backgrounds. Like other sectors where AI is making a huge impact, in AIEd this raises an important ethical question regarding giving students the freedom to opt out of AI powered predictions and automated evaluations.

The ethical implications of AI in education are dependent on the kind of disruption AI is doing in the ed-tech sector. On the one hand, this can be at an individual level for example by recommending wrong learning materials to students, or it can collectively impact relationships between different stakeholders such as how teachers perceive learners’ progress. This can also lead to automation bias and issues of accountability [67] where teachers begin to blindly rely on AI tools and prefer the tool’s outcomes over their own better judgement, whenever there is a conflict.

Initiatives have been observed in this space. For example, Professor Rose Luckin, professor of learner centered design at University College London along with Sir Anthony Seldon, vice chancellor of the University of Buckingham and Priya Lakhani, founder and CEO of Century Tech founded the Institute of Ethical AI in Education (IEAIEd) [72] to create awareness and promote the ethical aspects of AI in education. In its interim report, the institute identified seven different requirements for ethical AI to mitigate any kind of risks for learners. This included human agency and oversight to double-check AI’s performance, technical robustness and safety to prevent AI going wrong with new data or being hacked; diversity to ensure similar distribution of different demographics in data and avoid bias; non-discrimination and fairness to prevent anyone from being unfairly treated by AI; privacy and data governance to ensure everyone has the right to control their data; transparency to enhance the understanding of AI products; societal and environmental well-being to ensure that AI is not causing any harm and accountability to ensure that someone takes the responsibility for any wrongdoings of AI. Recently, the institute has also published a framework [71] for educators, schools and ed-tech companies to help them with the selection of ed-tech products with various ethical considerations in mind, like ethical design, transparency, privacy etc.

With the focus on online learning during the pandemic, and more utilization of AI powered ed-tech tools, risks of AI going wrong have increased significantly for all the stakeholders including ed-tech companies, schools, teachers and learners. A lot more work needs to be done on ethical AI in learning contexts to mitigate these risks, including assessment balancing risks and opportunities.

UNESCO published ‘Beijing Consensus’ on AI and Education that recommended member states to take a number of actions for the smooth and positively impactful integration of AI with education [74]. International bodies like EU have also recently published a set of draft guidelines under the heading of EU AI Act to ban certain uses of AI and categorize some as ‘high risk’ [47].

## Future work

With the focus on online education due to Covid’19 in the past year, it will be consequential to see what AI has to offer for education with vast amounts of data being collected online through Learning Management Systems (LMS) and Massive Online Open Courses (MOOCS).

With this influx of educational data, AI techniques such as reinforcement learning can also be utilized to empower ed-tech. Such algorithms perform best with the large amounts of data that was limited to very few ed-tech companies in 2021. These algorithms have achieved breakthrough performance in multiple domains including games [66], healthcare [14] and robotics [34]. This presents a great opportunity for AI’s applications in education for further enhancing students’ learning outcomes, reducing teachers’ workloads [30] and making learning personalized [64], interactive and fun [50, 53] for teachers and students.

With a growing number of AI powered ed-tech products in future, there will also be a lot of research on ethical AIEd. The risks of AI going wrong in education and the psychological impact this can have on learners and teachers is huge. Hence, more work needs to be done to ensure robust and safe AI products for all the stakeholders.

This can begin from the ed-tech companies sharing detailed guidelines for using AI powered ed-tech products, particularly specifying when not to rely on them. This includes the detailed documentation of the entire machine learning development pipeline with the assumptions made, data processing approaches used and the processes followed for selecting machine learning models. Regulators can play a very important role in ensuring that certain ethical principles are followed in developing these AI products or there are certain minimum performance thresholds that these products achieve [32].

## Conclusion

AIEd promised a lot in its infancy around 3 decades back. However, there are still a number of AI breakthroughs required to see that kind of disruption in education at scale (including basic infrastructure). In the end, the goal of AIEd is not to promote AI, but to support education. In essence, there is only one way to evaluate the impact of AI in Education: through learning outcomes. AIEd for reducing teachers’ workload is a lot more impactful if the reduced workload enables teachers to focus on students’ learning, leading to better learning outcomes.

Cutting edge AI by researchers and companies around the world is not of much use if it is not helping the primary grade student in learning. This problem becomes extremely challenging because every learner is unique with different learning pathways. With the recent developments in AI, particularly reinforcement learning techniques, the future holds exciting possibilities of where AI will take education. For impactful AI in education, learners and teachers always need to be at the epicenter of AI development.
